# Temporal development of the gut microbiome in early childhood from the TEDDY study

**DOI:** 10.1038/s41586-018-0617-x

**Published:** 2018-10-24

**Authors:** Christopher J. Stewart, Nadim J. Ajami, Jacqueline L. O’Brien, Diane S. Hutchinson, Daniel P. Smith, Matthew C. Wong, Matthew C. Ross, Richard E. Lloyd, HarshaVardhan Doddapaneni, Ginger A. Metcalf, Donna Muzny, Richard A. Gibbs, Tommi Vatanen, Curtis Huttenhower, Ramnik J. Xavier, Marian Rewers, William Hagopian, Jorma Toppari, Anette-G. Ziegler, Jin-Xiong She, Beena Akolkar, Ake Lernmark, Heikki Hyoty, Kendra Vehik, Jeffrey P. Krischer, Joseph F. Petrosino

**Affiliations:** 1 Alkek Center for Metagenomics and Microbiome Research, Department of Molecular Virology and Microbiology, Baylor College of Medicine, Houston, TX USA; 2 Institute of Cellular Medicine, Newcastle University, Newcastle upon Tyne, UK; 3 Human Genome Sequencing Center, Baylor College of Medicine, Houston, TX USA; 4 Broad Institute of MIT and Harvard, Cambridge, MA USA; 5 Barbara Davis Center for Childhood Diabetes, University of Colorado, Aurora, CO USA; 6 Pacific Northwest Research Institute, Seattle, WA USA; 7 Institute of Biomedicine, Research Centre for Integrative Physiology and Pharmacology, University of Turku, Turku, Finland; 8 Department of Pediatrics, Turku University Hospital, Turku, Finland; 9 Institute of Diabetes Research, Helmholtz Zentrum München, Munich, Germany; 10 Forschergruppe Diabetes, Technische Universität München, Klinikum Rechts der Isar, Munich, Germany; 11 Forschergruppe Diabetes e.V. at Helmholtz Zentrum München, Munich, Germany; 12 Center for Biotechnology and Genomic Medicine, Medical College of Georgia, Augusta University, Augusta, GA USA; 13 National Institute of Diabetes & Digestive & Kidney Diseases, Bethesda, MD USA; 14 Department of Clinical Sciences, Lund University/CRC, Skane University Hospital, Malmö, Sweden; 15 Department of Virology, Faculty of Medicine and Biosciences, University of Tampere, Tampere, Finland; 16 Fimlab Laboratories, Pirkanmaa Hospital District, Tampere, Finland; 17 Health Informatics Institute, Morsani College of Medicine, University of South Florida, Tampa, FL USA

**Keywords:** Microbiome, Clinical microbiology

## Abstract

The development of the microbiome from infancy to childhood is dependent on a range of factors, with microbial–immune crosstalk during this time thought to be involved in the pathobiology of later life diseases^1–9^ such as persistent islet autoimmunity and type 1 diabetes^10–12^. However, to our knowledge, no studies have performed extensive characterization of the microbiome in early life in a large, multi-centre population. Here we analyse longitudinal stool samples from 903 children between 3 and 46 months of age by 16S rRNA gene sequencing (*n* = 12,005) and metagenomic sequencing (*n* = 10,867), as part of the The Environmental Determinants of Diabetes in the Young (TEDDY) study. We show that the developing gut microbiome undergoes three distinct phases of microbiome progression: a developmental phase (months 3–14), a transitional phase (months 15–30), and a stable phase (months 31–46). Receipt of breast milk, either exclusive or partial, was the most significant factor associated with the microbiome structure. Breastfeeding was associated with higher levels of *Bifidobacterium* species (*B. breve* and *B. bifidum*), and the cessation of breast milk resulted in faster maturation of the gut microbiome, as marked by the phylum Firmicutes. Birth mode was also significantly associated with the microbiome during the developmental phase, driven by higher levels of *Bacteroides* species (particularly *B*. *fragilis*) in infants delivered vaginally. *Bacteroides* was also associated with increased gut diversity and faster maturation, regardless of the birth mode. Environmental factors including geographical location and household exposures (such as siblings and furry pets) also represented important covariates. A nested case–control analysis revealed subtle associations between microbial taxonomy and the development of islet autoimmunity or type 1 diabetes. These data determine the structural and functional assembly of the microbiome in early life and provide a foundation for targeted mechanistic investigation into the consequences of microbial–immune crosstalk for long-term health.

## Abstract (web summary)

Metagenomic sequencing analysis of stool samples from 903 children as part of the TEDDY study shows that breastfeeding was the most important factor associated with microbiome structure, and the cessation of breast milk resulted in faster maturation of the gut microbiome.

## Main

In this study, a total of 12,500 stool samples from 903 children from three European countries (Germany, Sweden and Finland) and three US states (Colorado, Georgia and Washington) were analysed. The children represent those who seroconverted to islet cell autoantibody positivity or developed type 1 diabetes (T1D) and matched controls. Stool samples were collected, on average, monthly from around 3 months of age as part of the The Environmental Determinants of Type 1 Diabetes in the Young (TEDDY) study^13^. After rarefaction and limiting samples to 3–46 months of age, we analysed the microbiome (16S rRNA gene sequencing, *n* = 12,005 samples from 903 children; metagenomic sequencing, *n* = 10,867 samples from 783 children) and functional metagenome (metagenomic sequencing only) from longitudinal stool samples **(**Extended Data Table 1). A companion paper by Vatanen et al.^14^ focused exclusively on metagenomic sequencing data.

In this cohort of children that are at-risk for developing islet autoimmunity (IA) or T1D, we aimed to (1) characterize definitively the longitudinal gut microbiome development from 3 to 46 months of age; (2) determine selected maternal and postnatal influences on the developing bacterial community during this same time period of early development; and (3) use a nested case–control analysis to investigate the potential of the microbiome as a predictor for the development of IA or T1D.

**Fig. 1:**
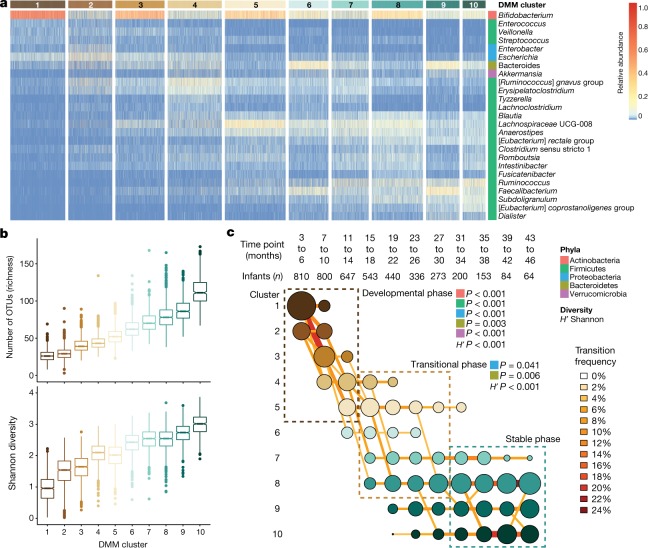
DMM clustering of 16S rRNA gene sequencing data (*n* = 12,005). The entire dataset formed ten distinct clusters based on lowest Laplace approximation. **a**, Heat map showing the relative abundance of the 25 most dominant bacterial genera per DMM cluster. Taxa names in square brackets are in need of formal taxonomic revision. **b**, Box plots showing the alpha diversity (richness and Shannon’s diversity) per each DMM cluster. The centre line denotes the median, the boxes cover the 25th and 75th percentiles, and the whiskers extend to the most extreme data point, which is no more than 1.5 times the length of the box away from the box. Points outside the whiskers represent outlier samples. **c**, Transition model showing the progression of samples through each DMM cluster per each time point, from months 3 to 46 of life. Dashed boxes show the three phases of microbiome progression (developmental, transitional and stable phase). Solid squares next to the labels denote the significant changes in phyla and Shannon’s diversity (*H*') per phase based on multiple linear regression. All phyla and the *H*' were significant in the developmental phase, two phyla and the *H*' were significant in the transitional phase, and no phyla or the *H*' were in the stable phase. Nodes and edges are sized based on the total counts. Nodes are coloured according to DMM cluster number and edges are coloured by the transition frequency. Transitions with less than 4% frequency are not shown. Results are further supported by the metagenomic sequencing data in Extended Data Fig. 2.

**Extended Data Fig. 1:**
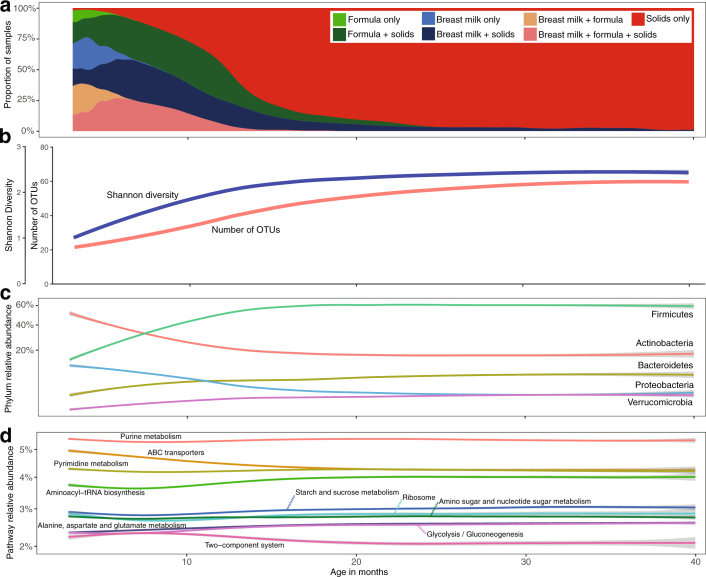
Characterization of the gut microbiome over the first 40 months of life (*n* = 11,717). **a**–**d**, 16S rRNA gene sequencing (**a**–**c**) and metagenomic sequencing (**d**) analysis. Curves show LOESS fit for the data per category, and shaded areas show permutation-based 95% confidence intervals for the fit. **a**, Summary of overall dietary status. **b**, The mean alpha diversity (richness and Shannon diversity) per child increased rapidly from 3 to 20 months of life. **c**, The mean relative abundance of the five most abundant bacterial phyla show changes from 3 to 20 months of life and generally remain stable after month 30 of life. **d**, The mean relative abundance of the ten most abundant bacterial pathways shows relative stability, with ABC transporters and two-component system showing the largest reduction from 3 to 20 months of life.

**Extended Data Fig. 2:**
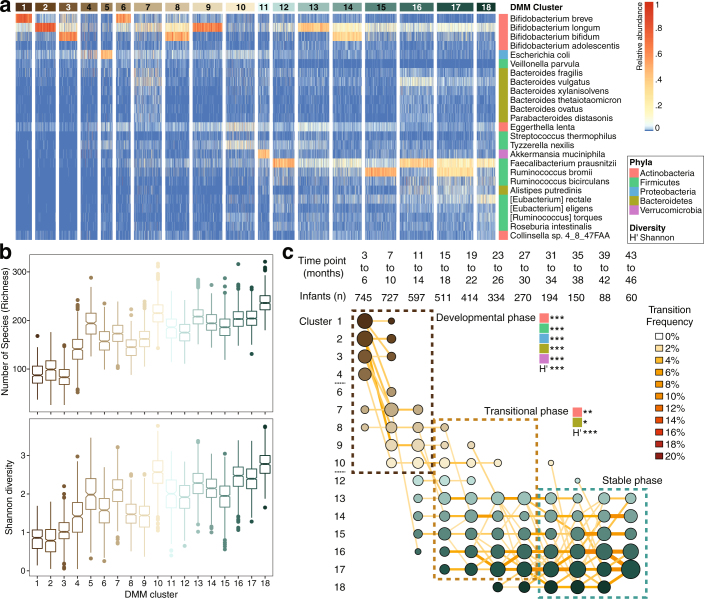
DMM clustering of metagenomic sequencing data (*n* = 10,867). The entire dataset formed 18 distinct clusters based on lowest Laplace approximation. **a**, Heat map showing the relative abundance of the 25 most dominant bacterial species per each DMM cluster. **b**, Box plots showing the alpha diversity (richness and Shannon’s diversity) for each DMM cluster. The centre line shows the median, the boxes cover the 25th and 75th percentiles, and the whiskers extend to the most extreme data point, which is no more than 1.5 times the length of the box away from the box. Points outside the whiskers represent outlier samples. **c**, Transition model showing the progression of samples through each DMM cluster per each time point, from months 3 to 46 of life. Dashed boxes show the three phases of microbiome progression (developmental, transitional and stable phase). Solid squares next to the labels denote the significant changes in phyla and Shannon diversity (*H*') per phase based on multiple linear regression. All phyla and the *H*' were significant in the developmental phase, two phyla and the *H*' were significant in the transitional phase, and no phyla or the *H*' were in the stable phase. Nodes and edges are sized based on the total counts. Nodes are coloured according to DMM cluster number and edges are coloured by the transition frequency. Transitions with less than 2% frequency were omitted from the plot.

A general overview of bacterial taxonomic and functional pathway development is provided in Supplementary Note 1 and Extended Data Fig. 1. Dirichlet multinomial mixtures (DMM) modelling was applied to 16S rRNA gene sequencing (Fig. 1) and metagenomic sequencing data (Extended Data Fig. 2). All samples from 3 to 46 months of age were included, and 16S rRNA gene sequencing profiles formed ten clusters (based on lowest Laplace approximation) (Fig. 1a). Bacterial richness and diversity increased in each cluster (Fig. 1a, b). Using linear mixed-effects modelling of the top five phyla and Shannon’s diversity index, we determined three distinct phases of microbiome progression: a developmental phase (months 3–14), a transitional phase (months 15–30), and a stable phase (≥31 months), in which all five phyla and the Shannon diversity index changed significantly during the developmental phase, two phyla (*Proteobacteria* and *Bacteroidetes*) and the Shannon diversity index changed significantly during the transitional phase, and all phyla and the Shannon diversity index were unchanged during the stable phase (Fig. 1c). *Bifidobacterium* dominated during the initial developmental phase, in which 20% of individuals transitioned from cluster 1 to cluster 3 (*Bifidobacterium* was dominant in both clusters). As infants aged, the microbiomes of their stools diversified into clusters 4–8 during months 15–30 (that is, the transitional phase). Microbiome stabilization, in which infants’ samples remained in the same cluster at consecutive time points, was observed from month 31 of life. Clusters 8–10 were the most dominant during the stable phase, with these clusters characterized by high alpha diversity and dominance of genera within the Firmicutes phyla. The three microbiome phases and changes in taxa are consistent with other cohorts^15–18^ and were supported by the metagenomic sequencing data (Supplementary Note 2 and Extended Data Fig. 2).

**Fig. 2:**
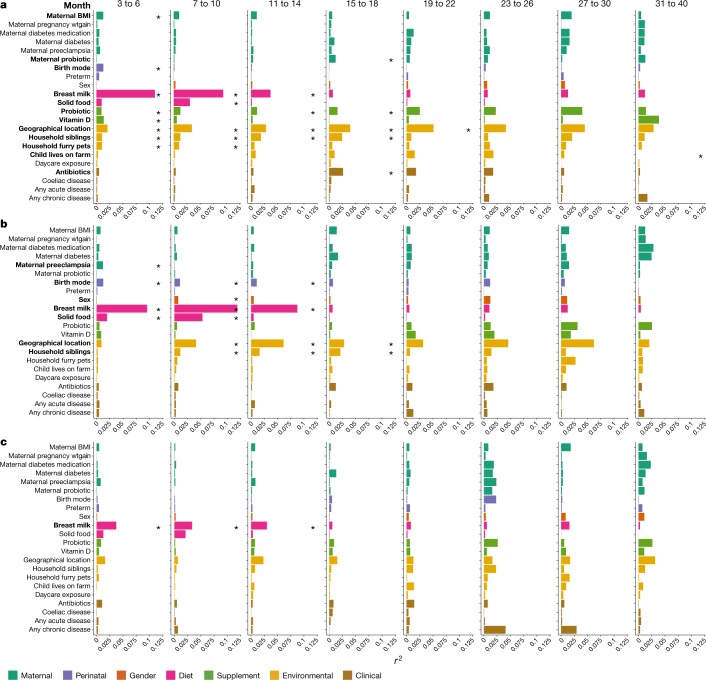
Significance and explained variance of 22 microbiome covariates modelled by EnvFit across all data types. Horizontal bars show the amount of variance (*r*^2^) explained by each covariate in the model as determined by EnvFit. The groups within each covariate are detailed in Extended Data Table 1. Covariates are coloured based on overall metadata group. Significant covariates (false discovery rate (FDR) *P* < 0.05) are represented in bold font. Asterisk denotes the significant covariates at each time point. BMI, body mass index; wtgain, weight gain. **a**, Microbiome profiles at the genus level based on 16S rRNA gene sequencing data (*n* = 4,069). **b**, Microbiome profiles at the species level based on metagenomic sequencing (*n* = 3,843). **c**, Functional metagenomic capacity at the module level based on metagenomic sequencing (*n* = 3,843).

We next sought to determine the significant factors associated with the microbiome profiles from 16S rRNA gene sequencing (genus level), metagenomic sequencing taxa (species level), and functional metabolic capacity (Kyoto encyclopedia of genes and genomes (KEGG) modules) (Supplementary Table 1). For statistical analysis, covariates were analysed by stratifying the samples into discrete time points (months 3–6, 7–10, 11–14, 15–18, 19–22, 23–26, 27–30 and 31–40), and only the first sample from each infant was included. Information about the underlying grouping of each covariate is shown in Extended Data Table 1. Several covariates were significantly associated with the genus and species level bacterial community profiles between months 3 and 18 of age, particularly at the first time point of 3 to 6 months (Fig. 2). Conversely, bacterial metabolic potential was associated exclusively with the consumption of breast milk from months 3 to 14 of life (Fig. 2).

**Fig. 3:**
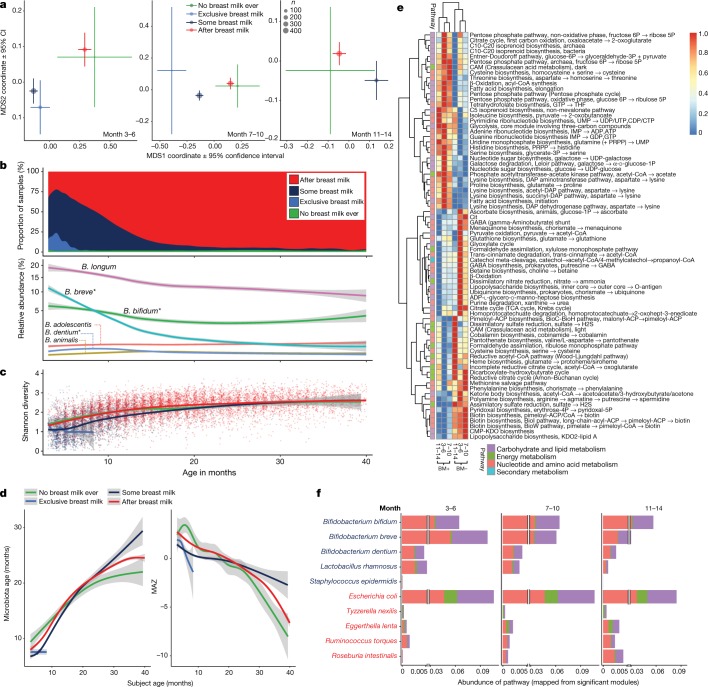
Breastfeeding status was the most significant microbiome covariate associated with all datasets throughout the first year of life. Breastfeeding status was significantly associated with microbiome profiles over the first three time points (months 3–14, *n* = 2,257; Supplementary Table 1). Curves show locally weighted scatterplot smoothing (LOESS) for the data per category, and shaded areas show permutation-based 95% confidence intervals for the fit. **a**, Non-metric multidimensional scaling (NMDS) ordination plots showing the mean centroid of each breastfeeding status group. Plots include only the first sample obtained from a patient within a given time point; months 3–6, 7–10 and 11–14. Centroid size based on number of samples and the bars represent the ±95% confidence interval. **b**, Plots showing the receipt of breast milk from months 3 to 40 of age compared to the relative abundance of the six most abundant *Bifidobacterium* species over the same period (*n* = 11,717). **c**, Longitudinal Shannon diversity index from months 3 to 40 of age (*n* = 11,717). **d**, Longitudinal development of the microbiome maturation based on the microbiota age and MAZ score against the age of the infant at sampling (*n* = 11,717). **e**, Heat map showing the mean abundance of all significant modules as determined by MaAsLin analysis at each of the first three time points. The corresponding pathway for each module is also presented. BM, breast milk. **f**, Stacked bar plots showing the abundance of each significant module binned at the pathway level. Abundance plotted per bacterial species, with the five most significant species associated with breastfed and non-breastfed infants, respectively.

Breastfeeding explained the greatest amount of variance from months 3 to 14 of life, after which only 10% of infants received any breast milk (Fig. 2). Breastfeeding had a comparable influence on microbiome development, regardless of whether it was exclusive or together with formula milk and/or solids (Fig. 3a). At the genus level, the receipt of breast milk was most significantly associated with *Bifidobacterium* throughout each time window (Supplementary Table 2). At the species level, breastfeeding was significantly associated with 121 different bacterial species, with higher levels of *B. bifidum*, *B. breve*, *B. dentium*, *Lactobacillus rhamnosus* and *Staphylococcus epidermidis*, and lower levels of *Escherichia coli*, *Tyzzerella nexilis*, *Eggerthella lenta*, *Ruminococcus torques* and *Roseburia intestinalis* in infants that were breastfed (a full list of significant taxa and associated *P* values are shown in Supplementary Table 2). *Bifidobacterium* spp. and *Lactobacillus* spp. exist viably in breast milk and *Staphylococcus* spp. colonize the areolar skin, thus these species can be directly transferred from the mother to infant^19–22^. *B. longum* was not significantly associated with breastfeeding and remained in higher relative abundance compared to other *Bifidobacterium* spp. (Fig. 3b). In the companion manuscript by Vatanen et al.^14^, most *B. longum* strains were found to contain genes from the human milk oligosaccharide (HMO) gene cluster, whereas after the cessation of breast milk, most *B. longum* strains no longer carried these genes. This potentially reflects the ability of *B. longum* subsp. *infantis* and subsp. *longum* to use mammalian- and plant-derived oligosaccharides, respectively^23,24^. *B. bifidum* also persisted after the cessation of breastfeeding, and this species is able to switch HMO to mucin degradation^24^. Vatanen et al.^14^ show experimentally that *B. breve*, *B. longum* and *B. bifidum*, which make up DMM clusters 1–3 (Extended Data Fig. 2), have distinct profiles of sugar utilization, suggesting that the different nutrient availability between infants can promote the colonization of specific *Bifidobacterium* species.

**Extended Data Fig. 3:**
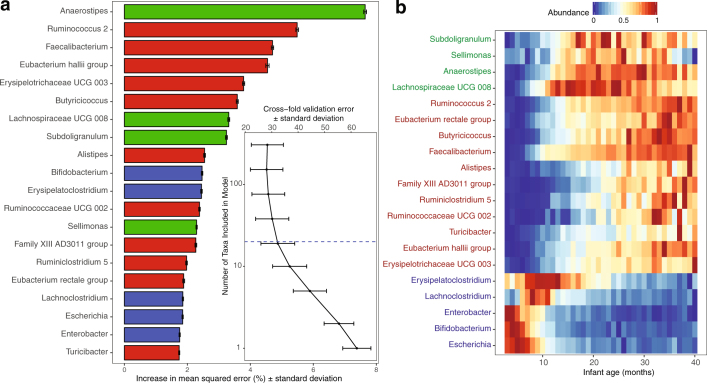
Twenty bacterial OTUs classified by random forest regression analysis as most age discriminatory over the first 40 months of life. Rank importance of OTUs determined by applying the random forest regression to the chronological age of 150 full-term, vaginally delivered, breastfed infants (*n* = 2,871 stool samples). The importance of OTUs is determined by the percentage increase in mean-squared error of microbiota age prediction when the relative abundance of each OTU were randomly permuted (mean importance ± s.d., *n* = 100 replicates). These selected OTUs explained 72% of the variance (compared to 75% variance explained with all OTUs in model) and were used to define maturation of the gut microbiome by microbiota age and MAZ score. OTUs are named to the genus level and coloured based on association with life stage; blue were associated with samples collected in the first 15 months, green with samples collected between months 15 and 30, and red were with samples collected after month 30. **a**, Twenty OTUs ranked by importance to the accuracy of the model. The tenfold cross-validation error is also displayed in order of variable importance. Blue dotted line represents the 20 OTUs used in the model. **b**, Heat map of mean relative abundance of the 20 selected OTUs per month from 3 to 40 months of age.

As the infant ages, the proportion of solid foods in the diet increases (and the amount of breast milk decreases)^21^. In the current study, the Shannon diversity index between infants receiving some breast milk and infants no longer receiving breast milk began to converge over time, probably as a result of a reduced proportion of breast milk in the diet and therefore less dominance of *Bifidobacterium* (Fig. 3c). Infants receiving some breast milk had significantly lower diversity when compared with infants no longer receiving breast milk across all phases (*P* < 0.001 for all phases), owing to the dominance of *Bifidobacterium* in infants receiving breast milk. To explore microbiome maturation further, we used microbiota age and microbiota-by-age *Z*-scores (MAZ) as previously described^25^, with a model of 20 operational taxonomic units (OTUs) that explained 72% of the variance (compared to 74% when including all OTUs in the model) (Extended Data Fig. 3). Comparably, the microbiota age and MAZ scores were significantly reduced in infants receiving some breast milk in the developmental and transitional phases (both *P* < 0.001 for microbiota age and MAZ scores), but converged in the stable phase (microbiota age *P* = 0.331 and MAZ score *P* = 0.196) (Fig. 3d). After the cessation of breast milk, 110 unique bacterial species (89 from the Firmicutes phylum) were significantly increased from months 3 to 14 of life alone (Supplementary Table 2). The suppression of Firmicutes while in receipt of some breast milk was recently noted^21^. Together, these data support existing reports that the maturation of the gut microbiome is driven by the cessation of breast milk (rather than the introduction of solid foods), hallmarked by increased levels of Firmicutes^17,21,26^.

Breastfeeding was the only covariate that was significantly associated with metabolic potential (Fig. 2). Plotting all significant modules (Supplementary Table 1) from the first three time points (months 3–14) showed clear clustering based on the receipt of breast milk, with comparability in the metabolic capacity regardless of the time point (Fig. 3e). Modules most significantly associated with breastfed infants were from the ‘carbohydrate and lipid metabolism’ pathway and included ‘fatty acid biosynthesis’ (M00083 and M00082) and ‘beta-oxidation, acyl-CoA synthesis’ (M00086) (Supplementary Table 2). This is in accordance with previous work that found that genes that relate to the biosynthesis of fatty acids are increased during infancy in breastfed infants^17,27,28^. Conversely, infants not receiving breast milk showed rapid turnover of the metabolic capacity, and the ‘dicarboxylate-hydroxybutyrate cycle’ (M00374) and ‘reductive acetyl-CoA (Wood–Ljungdahl)’ (M00377) pathways were increased. Modules relating to vitamins B7 (‘nucleotide and amino acid metabolism’ pathway; M00573, M00577 and M00123) were also increased in all time points up to 14 months in non-breastfed infants, a function that is associated with the adult microbiome^28^.

By mapping reads with genomic coordinates that overlap with known KEGG orthologues to KEGG modules (M), we were able to directly determine from which taxa each gene orthology (and thus module) was derived (see Methods). Each pathway from which each significant module was derived was plotted against the main species discriminating breastfeeding status (Supplementary Table 2). In breastfed infants, *B. breve* accounted for the highest number of significant modules in early life, and was replaced by *B. bifidum* after 6 months of life (Fig. 3f). In non-breastfed infants, *E. coli* primarily accounted for the significant modules between 3 and 14 months of life (Fig. 3f). This provides further evidence that the gut microbiome rapidly matures after the cessation of breast milk, both at the taxonomic and functional levels.

**Extended Data Fig. 4:**
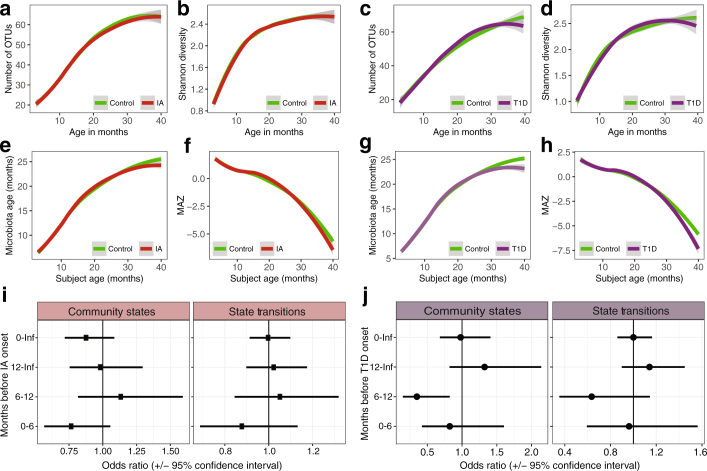
The microbiota was not associated with the development of persistent IA and T1D. Data are based on 16S rRNA gene sequencing (*n* = 11,717). Analysis based on a nested 1:1 case–control cohort of equal samples. Curves show LOESS fit for the data per category, and shaded areas show permutation-based 95% confidence intervals for the fit. **a**, **b**, The number of OTUs (**a**) and the Shannon’s diversity index (**b**) in the IA cohort. **c**, **d**, The number of OTUs (**c**) and Shannon’s diversity (**d**) in the T1D cohort. **e**, **f**, Microbiota age (**e**) and MAZ score (**f**) in the IA cohort. **g**, **h**, Microbiota age (**g**) and MAZ score (**h**) in the T1D cohort. **i**, **j**, Forest plot showing the odds ratios for the association between the microbiome stability metrics and development of IA (**i**) and T1D (**j**). A separate conditional logistic regression was run for four time intervals: (1) birth to onset; (2) 12 months before onset; (3) 6–12 months before onset; and (4) 6 months before onset. Models were adjusted for HLA genotype, mode of delivery, duration of exclusive breastfeeding, number of antibiotic courses, and number of infectious episodes. Community states are the total number of unique clusters exhibited by an infant and state transitions are the number of transitions between clusters. No odds ratio was significantly different between cases and controls (Supplementary Table 3).

The TEDDY study was powered to detect microbiome associations with the development of IA and T1D based on a specific 1:1 nested case–control study design, from two nested case–control studies (IA or T1D), using risk set sampling^29^. The analytical cohort consisted of a subset with an equal number of samples for each case–control pair. The IA cohort consisted of 632 children and 6,194 stool samples and the T1D cohort consisted of 196 children and 1,540 stool samples, as of 31 May 2012 (Supplementary Table 3). The temporal alpha diversity (both richness and Shannon’s diversity), microbiota age and MAZ scores were comparable between cases and matched controls for both the IA and T1D groups (all *P* > 0.05; Extended Data Fig. 4a–h). The relative abundance of the top 50 most abundant genera from 16S rRNA gene sequencing showed only subtle compositional differences, with higher relative abundance of an unclassified Erysipelotrichaceae (*P* = 0.019) in cases of IA (Supplementary Table 3). In the T1D and control cohort, five bacterial genera were associated with T1D onset, with *Parabacteroides* the most significant (*P* < 0.001). Eleven bacterial genera were lower in T1D cases, including four unclassified Ruminococcaceae, *Lactococcus* (*P* = 0.020), *Streptococcus* (*P* = 0.032), and *Akkermansia* (*P* = 0.045) (Supplementary Table 3).

Conditional logistic regression models showed no significant associations between either the numbers of unique states exhibited or the number of transitions between different states per subject for IA (Extended Data Fig. 4i and Supplementary Table 3). The lack of associations was consistent in T1D, with the exception that cases exhibited fewer unique states 6–12 months before the onset of T1D (*P* = 0.032) (Extended Data Fig. 4j and Supplementary Table 3). Notably, the 6–12 months before T1D onset group consisted of the lowest number of samples for any of the time points (*n* = 67 subjects per group), and thus the statistically significant result should be interpreted with caution. Overall, the conditional logistic regression models of community dynamics suggest that microbiome stability was not strongly related to the onset of IA or T1D.

**Extended Data Fig. 5:**
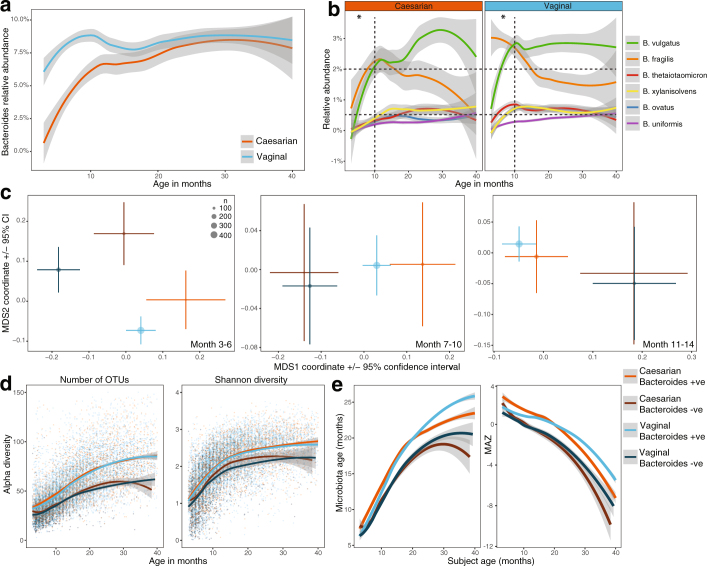
Association of the gut microbiome with birth mode. Birth mode was significantly associated with the microbiome in months 3–6 by 16S rRNA gene sequencing and in all time points up to month 14 by metagenomic sequencing (see Supplementary Table 1). Curves show LOESS fit for the data per category, and shaded areas show permutation-based 95% confidence intervals for the fit. **a**, Longitudinal development of the *Bacteroides* genus as determined by 16S rRNA gene sequencing (*n* = 11,717). **b**, Longitudinal development of the six most abundant species within the *Bacteroides* genera as determined by metagenomic sequencing (*n* = 10,867). Grid overlay added to aid visual interpretation. **c**, NMDS ordination plots showing the mean centroid of each birth mode group stratified by *Bacteroides* positive or negative based on detection 16S rRNA gene sequencing. Plots include only the first sample obtained from a patient within a given time point for months 3–6, 7–10 and 11–14 (*n* = 2,257). Centroid size based on number of samples and the bars represent the ±95% confidence interval. **d**, Longitudinal development of the alpha diversity (richness and Shannon’s diversity) with birth mode further stratified according to *Bacteroides* positive or negative (*n* = 11,717). **e**, Longitudinal development of the microbiome maturation based on the microbiota age and MAZ score against the age of the infant at sampling (*n* = 11,717). Birth mode was further stratified according to *Bacteroides* positive or negative.

**Extended Data Fig. 6:**
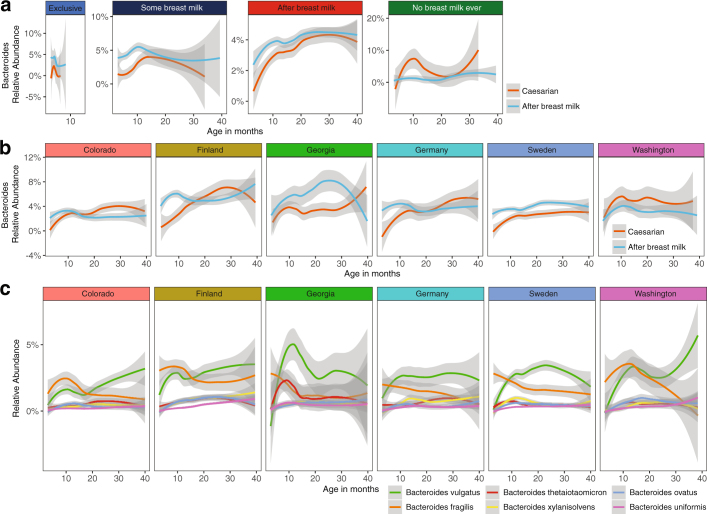
The relative abundance of *Bacteroides* stratified by breast milk and geographical location. Curves show LOESS fit for the data per category, and shaded areas show permutation-based 95% confidence intervals for the fit. **a**, **b**, *Bacteroides* genera based on 16S rRNA gene sequencing data (*n* = 11,717) stratified by breast milk status (**a**) and geographical location (**b**). **c**, The top 6 *Bacteroides* species based on metagenomic sequencing data (*n* = 10,867) stratified by geographical location.

**Extended Data Fig. 7:**
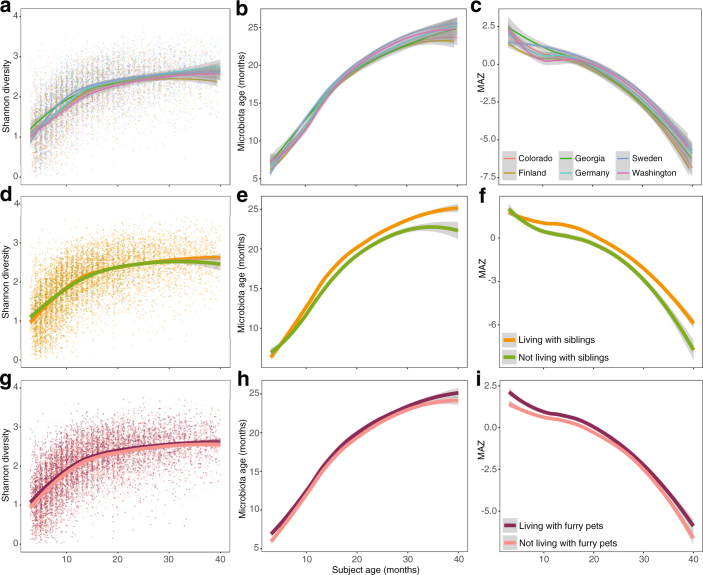
Environmental covariates significantly associated with the microbiome profiles. 16S rRNA gene sequencing data plotted from months 3–40 of life (*n* = 11,717). Curves show LOESS fit for the data per category, and shaded areas show permutation-based 95% confidence intervals for the fit. Significance determined by linear mixed-effects models in accordance with observed phases of maturation: developmental (months 3–14), transitional (months 15–30), and stable (months 31–46). Shaded lines represent the ±95% confidence interval. Longitudinal development of the Shannon’s diversity index, microbiota age and MAZ score by geographical location (**a**–**c**), occurrence of household siblings (**d**–**f**), and occurrence of household furry pets (**g**–**i**).

Further analysis of covariates that were significant at several time points and/or consistently significant by 16S rRNA gene sequencing and metagenomics are presented in Supplementary Note 3. In brief, birth mode was significantly associated with microbiome development over the first year of life, with higher levels of *Bacteroides* spp. in infants that were delivered vaginally (Extended Data Fig. 5). This was generally consistent across the different breast milk exposure groups and geographical locations (Extended Data Fig. 6). Differences between geographical locations occurred from 3 to 22 months of life (Supplementary Table 1), although the core microbiome was consistent (Supplementary Table 4), and diversity, microbiota age and MAZ scores had comparable trajectories across each location (Extended Data Fig. 7a–c). Household exposures (for example, living with siblings and with furry pets) were also associated with differences in the microbiome profiles in early life, in which infants living with siblings and/or with furry pets showed accelerated rates of maturation of the microbiome (Extended Data Fig. 7d–i).

The TEDDY population offers a robust analysis of gut microbiome development of 903 infants from months 3 to 46 of age, with regular sampling (more than 12,000 stool samples), extensive metadata, and the use of both amplicon and metagenomic sequencing. We showed that the first year of life is a key phase for the development of the microbiome, with the receipt of breast milk being the main factor that influences microbiome development over this period. Birth mode, geographical location, household siblings and furry pets were also associated with the microbiome over this period. We considered the first year of life as developmental, the second year of life as transitional, and from year three of life the microbiome stabilized. These precise ages may shift when investigators include samples before month 3 or beyond month 46 of life.

The current cohort is largely white, non-Hispanic and is drawn from a population of infants at high genetic risk for T1D, some of whom developed autoimmunity or diabetes. Temporal alpha diversity and community dynamics were comparable between cases and controls, which is in contrast to findings reported in other cohorts and may reflect the increased number of subjects and samples in the TEDDY cohort^11,12^. We found subtle changes in the relative abundance of bacterial genera between cases (IA and/or T1D) and matched controls. T1D cases showed higher levels of *Streptococcus* sp. and *Lactococcus* sp., which is consistent with the findings of Vatanen et al.^14^ in the companion paper. In accordance with previous work, the abundance of *Akkermansia* was also higher in controls in the current study, which may be indicative of enhanced gut integrity^10^.

The overall microbiome development and significant covariates are in concordance with previous reports in westernized populations, although caution should be exercised when extrapolating the findings from the TEDDY cohort of children with risk factors of developing T1D to the wider population. Nevertheless, the significant covariates reported in the current study have been independently linked to the risk of later life diseases such as obesity, asthma and allergy^1–8^. The current study provides several testable hypotheses of microbiome development in infancy, and it remains important to determine the potential mechanism of altered early life microbiome and the subsequent effect on immune development and functioning. With a more comprehensive understanding of the crucial early life phases and their effect on health and disease, lifestyles and therapeutics can be tailored to support optimal microbial–immune homeostasis.

## Methods

### Study population

The TEDDY Study is composed of six clinical research centres: three in the United States (Colorado, Georgia/Florida and Washington), and three in Europe (Finland, Germany and Sweden). Children enrolled are followed prospectively from three months to 15 years with study visits every three months until age 4 years and every three or six months thereafter depending on autoantibody positivity. Stool samples and associated metadata were collected as of 31 May 2012. Stool samples were collected monthly from 3 to 48 months of life, then every three months until the age of 10 years, and then biannually thereafter, into the three plastic stool containers provided by the clinical centre. Children who were antibody negative after 4 years of age were encouraged to submit four times a year even though after 4 years their visits schedule switched to biannual. Parents sent the stool containers at either ambient or +4 °C temperature with guaranteed delivery within 24 h in the appropriate shipping box to the NIDDK repository if living in the United States or their affiliated clinical centre if living in Europe. The European clinical centres stored the stool samples and sent monthly bulk shipments of frozen stool to the NIDDK repository. The population (both cases and controls) is based on children at high risk for T1D based on their HLA genotype with 10% based on family history in addition to HLA. Detailed study design and methods have been previously published^13,29,30^. Matching factors for case and control children were geographical location, sex and family history of T1D.

Metadata were collected using validated questionnaires that have been either published or extensively scrutinized by experts. Information about mothers, pregnancy and birth was collected during the three month clinic visit by questionnaire and included the mode of birth (vaginal birth versus Caesarean section), the infant’s 5-min Apgar score, pregnancy complications, information about maternal diabetes (T1D, type 2 diabetes (T2D) or gestational diabetes), gestational age, and maternal medication use (insulin, metformin, glyburide, antihypertensives) during pregnancy. TEDDY provides many tools, such as ‘The TEDDY book’, to the parents to assist in real-time collection of all events in their child’s life to ensure bias and error are minimized. At each visit the study personnel will go over the TEDDY book with the primary caretaker and extract pertinent information using standardized study forms. Data are extracted by trained staff members during scheduled visits every three months starting at 3 months of age and entered directly via stand forms (web forms or teleforms), which are transmitted electronically. Front-end constraints are used in the web application to prevent the entry of invalid data and The TEDDY Error Reporting and Verification System (ERVS) consists of a set of programs that conduct automated quality control on the data, report and resolve errors, an integrated database for storing error data, and a set of programs that generate reports for monitoring data cleaning efforts. The details of the system have been published^31^. Given the prospective nature of the TEDDY design, information and recall bias are greatly minimized. Because the children do not have event outcome at time of enrolment and are followed, there is no reason for any systematic differences between groups of the study participants in the accuracy of the information collected.

The TEDDY study was approved by local US Institutional Review Boards and European Ethics Committee Boards in Colorado’s Colorado Multiple Institutional Review Board, Georgia’s Medical College of Georgia Human Assurance Committee (2004–2010), Georgia Health Sciences University Human Assurance Committee (2011–2012), Georgia Regents University Institutional Review Board (2013–2015), Augusta University Institutional Review Board (2015–present), Florida’s University of Florida Health Center Institutional Review Board, Washington state’s Washington State Institutional Review Board (2004–2012) and Western Institutional Review Board (2013–present), Finland’s Ethics Committee of the Hospital District of Southwest Finland, Germany’s Bayerischen Landesärztekammer (Bavarian Medical Association) Ethics Committee, Sweden’s Regional Ethics Board in Lund, Section 2 (2004–2012) and Lund University Committee for Continuing Ethical Review (2013–present). All parents or guardians provided written informed consent before participation in genetic screening and enrolment. The study was performed in compliance with all relevant ethical regulations.

A priori power calculations using discrete Cox’s proportional hazards regression^32^ for the matched IA case–control study estimated 80% power, *α* = 0.01, two-sided test to detect an odds ratio > 3 for an exposure with 5% prevalence to an odds ratio > 1.8 for an exposure with 20% prevalence. The experiments were not randomized, and investigators were not blinded to allocation during experiments and outcome assessment.

### 16S rRNA gene sequencing

16S rRNA gene sequencing methods were adapted from the methods developed by the NIH-Human Microbiome Project and the Earth Microbiome Project^33–35^. Bacterial DNA was extracted using the PowerMag Microbiome DNA isolation kit following the manufacturer’s instructions. The V4 region of the 16S rRNA gene was amplified by PCR and sequenced on the MiSeq platform (Illumina) using the 2 × 250 bp paired-end read protocol. The read pairs were demultiplexed and reads were merged using USEARCH v7.0.1090^36^. Merging allowed zero mismatches and a minimum overlap of 50 bases, and merged reads were trimmed at the first base with a *q* ≤ 5. A quality filter was applied to the resulting merged reads and those containing above 0.5% expected errors were discarded. Sequences were stepwise clustered into OTUs at a similarity cut-off value of 97% using the UPARSE algorithm^37^. Chimeras were removed using USEARCH v7.0.1090 and UCHIME v4.2. To determine taxonomies, OTUs were mapped to a version of the SILVA Database^38^ containing only the 16S V4 region using USEARCH v7.0.1090. Abundances were recovered by mapping the merged reads to the UPARSE OTUs. A custom script constructed a rarefied OTU table from the output files generated in the previous two steps for downstream analyses of taxonomic relative abundance, alpha diversity, and beta diversity (including UniFrac)^39^. A total of 114,313,601 reads (median 8,442 reads per sample) were obtained from 16S rRNA gene sequencing and each sample was rarefied to 3,000 reads. Stringent merging parameters account for the relatively low number of OTUs, with the number of species by metagenomics around fourfold higher than the number of OTUs by 16S rRNA gene sequencing.

### Metagenomic shotgun sequencing

Individual libraries constructed from each sample were pooled and loaded onto the HiSeq 2000 platform (Illumina) and sequenced using the 2 × 100 bp paired-end read protocol. The process of quality filtering, trimming, and demultiplexing was carried out by in-house pipeline developed by assembling publicly available tools such as Casava v1.8.2 (Illumina) for the generation of fastqs, Trim Galore v0.2.8 (http://www.bioinformatics.babraham.ac.uk/projects/trim_galore/) and cutadapt v1.9dev2 for adaptor and quality trimming, and PRINSEQ v0.20.5^40^ for sample dereplication and low complexity filtering. In addition, Bowtie2 v2.2.3^41^ was used to map reads to a database containing complete genomes and assemblies for bacteria, viruses, human, and vectors in the NCBI whole-genome sequencing (WGS) archive (as of March 2015). Reads in which the highest identity matches were not bacterial were removed from subsequent analysis. The edit distance (Levenshtein distance) was used to determine the score of the alignments to the reference genomes^42^. For bacterial reads, the highest scoring match (greater than 90%) was chosen per read considering only the top 25 highest scoring alignments. In the event of multiple identical top scoring hits, the lowest common ancestor was determined.

Reads in which the genomic coordinates overlap with known KEGG orthologues^43,44^ were tabulated, and KEGG modules were calculated step-wise and determined to be complete if 65% of the reaction steps were present per detected species and for the metagenome. Pathways were constructed for each taxa and metagenome by calculating the minimum set through MinPath^45^ resulting from the gene orthologues present. A total of 19,967,936,136 reads (median 1,606,240 reads per sample) were obtained from metagenomic sequencing and for subsequent analysis each sample was rarefied to 100,000 reads.

### Statistical analysis

The analysis was conducted in two parts: (1) characterize the longitudinal maturation of the microbiome and (2) determine the significant covariates that influence microbiome development. For both parts of analysis, alpha diversity (richness and Shannon diversity) was calculated at the OTU-level for 16S rRNA gene sequencing and species-level for metagenomics data. Alpha diversity and taxonomic abundance were modelled using LOESS regression, and implemented and plotted with 95% confidence intervals in R (http://www.R-project.org**)** using the ggplot package^46^.

#### DMM clustering

The first part of the analysis determined the key phases of microbiome progression, which included the use of DMM. DMM bins samples on the basis of microbial community structure^47^. The appropriate number of clusters was determined based on the lowest Laplace approximation score. For this specific analysis, samples up to month 46 of life were included, whereas all other analyses included samples up to month 40 of life. Including the additional samples here allowed for more accurate determination of the microbiome phases.

The second part of the analysis sought to determine the significant covariates in shaping the microbiome profiles at discrete time points and further ascertain the significantly altered taxa based on samples up to month 40 of life. The framework for the statistical analysis considered the longitudinal nature of the dataset and accounted for the dynamic nature of the covariates. Owing to the potential that some covariates might influence the microbiome before the start date (for example, underlying indication for an antibiotic prescription) and some covariates will alter the microbiome for an unknown time frame (for example, microbiome disrupted by antibiotics may continue to be altered months after treatment), covariates were classified as ‘before’, ‘during’, or ‘after’. In the case a covariate was negative for an infant, all samples would be classified as ‘never’. In instances in which several onsets of a covariate were possible (for example, multiple antibiotic start and end time points), after the first onset the covariate was classified as ‘after’ for the remaining samples, unless another event occurred, in which case ‘during’ would be applied where appropriate according to the start and stop dates. Analysis was performed at specific time windows, including samples collected between months 3–6, 7–10, 11–14, 15–18, 19–22, 23–26, 27–30 and 31–40. Only the first sample collected from a given child was included in each time window to account for repeated measures.

#### EnvFit analysis to determine significant covariates

The effect size and significance of each covariate were determined using the ‘envfit’ function in ‘vegan’ (https://cran.r-project.org/web/packages/vegan/index.html) comparing the difference in the centroids of each group relative to the total variation. Ordination was performed using NMDS based on Bray–Curtis dissimilarity. The significance value was determined based on 10,000 permutations. All *P* values derived from envfit were adjusted for multiple comparisons using FDR adjustment (Benjamini–Hochberg procedure)^48^. In total, 22 covariates with known associations to gut microbiome development in neonates, infants, and children were included in the envfit analysis and the grouping used for within each variable is presented in Extended Data Table 1. Specifically, we tested maternal factors including diabetes (gestational, T1D, T2D or none)^49^, diabetes medication (insulin, metformin, glyburide, antihypertensives)^50^, BMI^51,52^, gestational weight gain category (excess or non-excess)^53^, preeclampsia^52^, maternal probiotic consumption^54^, as well as offspring factors such as prematurity^18,55^, birth mode^15–17,56^, gender^56^, receipt of breast milk and/or formula^17,53,57–59^, introduction of solid foods^60,61^, geographical location^57^, probiotics^62^, vitamin D supplementation^63^, antibiotics^18^, household siblings^56,64^, household furry pets^64,65^, living on a farm with animals^66,67^, day-care exposure^68^, coeliac disease^69^, acute disease, and chronic disease^69^.

#### MaAsLin analysis to determine significant taxa associated with each covariate

MaAsLin was used for adjustment of covariates when determining the significance of taxa (genus level for 16S rRNA gene sequencing and species level for metagenomic sequencing) contributing to a specific variable, while accounting for potentially confounding covariates^70^. In brief, this multivariate linear modelling system for microbial data selects from among a set of (potentially high-dimensional) covariates to associate with microbial taxon or pathway abundances. Mixed-effects linear models using a variance-stabilizing arcsin square root transform on relative abundances are then used to determine the significance of putative associations from among this reduced set. Nominal *P* values across all associations are then adjusted using the Benjamini–Hochberg FDR method. Here, microbial features with corrected *q* < 0.25 were reported. All 22 covariates tested in the envfit were included in the adjustment regardless of significance by envfit. Subject age was also included to adjust for potential age driven changes in taxa within each three-month time window and IA and T1D outcome were included to adjust for the nested case control nature of the cohort. The default MaAsLin parameters were applied (maximum percentage of samples NA in metadata 10%, minimum percentage relative abundance 0.01%, *P* < 0.05, *q* < 0.25). All P values were adjusted for multiple comparisons using FDR^48^.

#### Microbiota maturation modelling and linear mixed-effects analysis

The random forest regression model^71^ was performed as previously described^25^, using the ‘randomForest’ R package^72^. In brief, the model was trained on 150 randomly selected full term (>37 weeks gestation), vaginally delivered, breastfed infants who had a minimum of 10 samples included in the final dataset. The model was built using the default parameters: growing 10,000 trees and *n*/3 OTUs randomly sampled at each split, in which *n* represents the number of OTUs. The model was further refined by applying ‘rfcv’ with tenfold cross-validation resulting in the inclusion of 20 OTUs to train the final model based on percentage increase in mean-squared error. These 20 OTUs explained 72% of the total variance of the model (compared to 75% with all OTUs included). The age of the subject predicted by this model was termed microbiota age and was further used to determine MAZ scores using the formulae described preiously^25^. Significant differences in alpha diversity, microbiota age, and MAZ scores were calculated using linear mixed-effects models in R, with the ‘lmer’ command within the ‘lme4’ package^73^. We included random slopes and intercept for individual children, and evaluated delivery mode, age, *Bacteroides* positive or negative, predominant diet, geographical location, presence of siblings, and presence of household pets as fixed effects. To perform these piecewise longitudinal models, we divided samples into the three developmental phases (<14 months, >15–<30 months, and >31 months). Owing to the relatively low number of samples in the exclusive and never breastfed groups, the analysis of breast milk status was conducted based on ‘some breast milk’ or ‘after breast milk’, with these groups found to cluster with exclusive and never breastfed, respectively.

#### Determination of the datasets for IA and T1D nested case–control stability analyses

The development of persistent confirmed IA was assessed every three months. Persistent autoimmunity was defined by the presence of confirmed islet autoantibody on two or more consecutive visits. The date of persistent autoimmunity was defined as the draw date of the first sample of the two consecutive samples that deemed the child persistent confirmed positive for a specific autoantibody (or any autoantibody). T1D was defined according to American Diabetes Association criteria for diagnosis^74^. A dataset with equal numbers of cases and control samples was created to preform conditional logistic regression of summary metric variables (that is, counting for each person the number of unique clusters exhibited and the number of temporal transitions between different clusters). On average, cases tended to have more samples than controls, and therefore had more transitions and observed states, which resulted in spurious associations between our metrics and disease outcome. For this purpose, we created a dataset in which case and control samples were matched to the paired case based on the nearest sample by day of life (unmatched sample or sample outside of ±20% were omitted from analyses). This resulted in an analytical cohort of 316 IA cases and 316 paired controls (*n* = 3,097 stool samples in each group) and 98 T1D cases and 98 paired controls (*n* = 1,270 stool samples in each group). For consistency, we used these datasets for all matched case–control analyses. The IA and T1D analysis was based on 16S rRNA gene sequencing data only and analysis of the metagenomic sequencing data (that is, species level taxonomic profiling and functional capacity) are presented in the companion paper^14^.

#### Taxonomic and metabolic profiling relative to IA onset in the matched case–control dataset

16S rRNA gene sequencing data was used to determine differences between alpha diversity (number of OTUs (richness) and Shannon’s diversity index), microbiota age, and MAZ scores. Significant differences in alpha diversity, microbiota age, and MAZ scores were calculated using linear mixed-effects models in R, with the ‘lmer’ command within the ‘lme4’ package^73^. To perform these piecewise longitudinal models, we divided samples into the three developmental phases (<14 months, >15–<30 months, and >31 months). Conditional logistic regression of matched case–control pairs was performed on the top 50 most dominant bacterial genera from samples prior to disease diagnosis. Odds ratios were calculated with 95% confidence intervals, adjusted for potential confounding variables, including age at sample collection, HLA genotype, mode of delivery, and duration of breastfeeding. Abundance information for genera was entered into the model as log_2_-transformed read counts. A value of 0.01 was added to avoid 0 s. The Benjamini–Hochberg procedure was applied to correct for multiple comparisons^48^ and corrected *P* < 0.05 was considered significant.

#### Assessment of microbiome instability based on DMM clusters between IA or T1D cases and controls

For each subject, the total number of clusters exhibited throughout sampling per infant and the number of transitions between different clusters from one sample to the next were calculated to provide summary measures of microbiome stability over time. These summary metrics were then used in conditional logistic regression to assess the relationship of microbiome stability with IA and T1D. Odds ratios were calculated with 95% confidence intervals, adjusted for potential confounding variables, including HLA genotype, mode of delivery, duration of breastfeeding, number of antibiotic courses, and number of infectious episodes.

### Reporting summary

Further information on research design is available in the Nature Research Reporting Summary linked to this paper.

### Code availability

Code for the transition model showing the progression of samples through each DMM cluster, which are presented in Fig. 1 and Extended Data Fig. 2, has been made publicly available at https://github.com/StewartLab/Stewart_TEDDY_Microbiome_Analysis. Other analysis software including quality control, taxonomic, and functional profilers is publicly available and referenced as appropriate.

## Online content

Any methods, additional references, Nature Research reporting summaries, source data, statements of data availability and associated accession codes are available at 10.1038/s41586-018-0617-x.

### Supplementary information

Supplementary Information This file contains Supplementary Notes 1-3, Supplementary References and a full list of members of the TEDDY Study Group

Reporting Summary

Supplementary Table 1 EnvFit P values for significant covariates determined by NMDS based on Bray-Curtis dissimilarity distance for each dataset.

Supplementary Table 2 MaAsLin P values for significant taxa for 16S rRNA gene sequencing (genus level taxa) and metagenomic sequencing (species level taxa and metabolic modules). Mixed effects linear models using a variance-stabilizing arcsin square root transform on relative abundances were used to determine the significance. P values were adjusted for multiple comparisons using the Benjamini-Hochberg false discovery rate (FDR) method.

Supplementary Table 3 Analytical population overview and results from each statistical analyses related to islet autoantibody and Type 1 Diabetes onset. P values based on conditional logistic regression of the nested case control cohort, with adjustment for age, human leukocyte antigen type, delivery mode, and breast milk feeding. P values were adjusted for multiple comparisons using the Benjamini-Hochberg false discovery rate (FDR) method.

Supplementary Table 4 Bacterial genera determined to be core microbiome (>90% prevalence) in the three microbiome phases between each of the geographical locations.

## Figures and Tables

**Extended Data Table 1:**
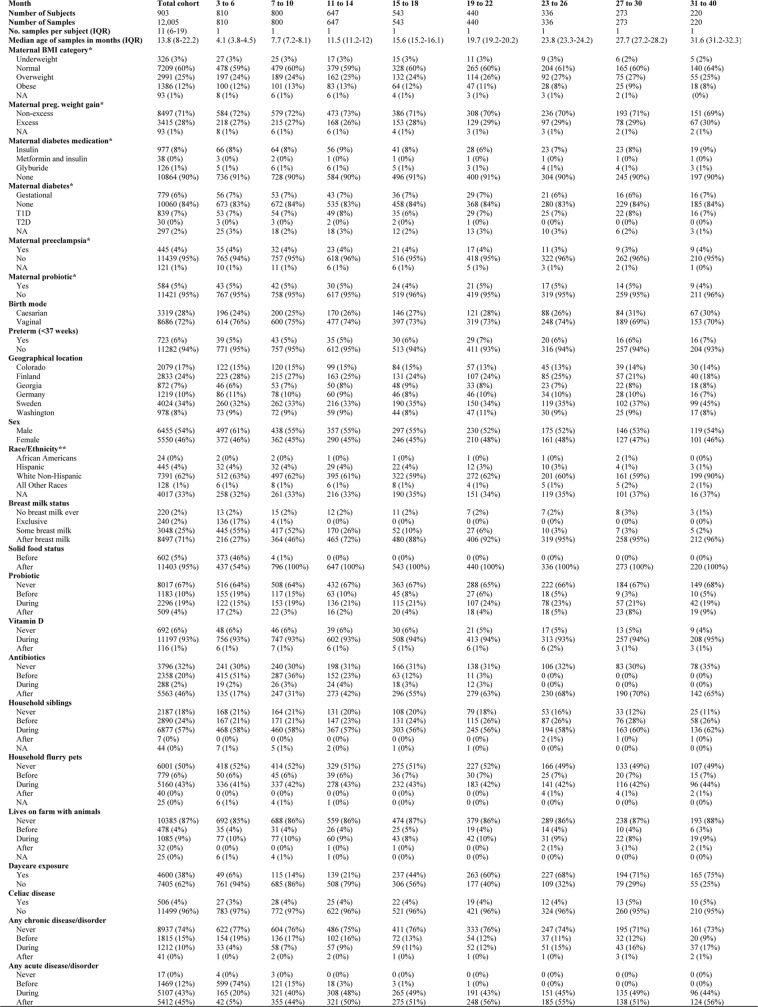
Overview of the entire analytical cohort

IQR, interquartile range; NA, not available.

*Maternal variables relate to measurements obtained during pregnancy.

**Race/ethnicity was only available for the US sites.

## Data Availability

TEDDY microbiome 16S rRNA gene sequencing and metagenomic sequencing data that support the findings of this study have been deposited in the NCBI database of Genotypes and Phenotypes (dbGaP) with the primary accession code phs001443.v1.p1, in accordance with the dbGaP controlled-access authorization process. Clinical metadata analysed during the current study will be made available in the NIDDK Central Repository at https://www.niddkrepository.org/studies/teddy.
