# Supplementary material for: Temporal development of the gut microbiome in early childhood from the TEDDY study
Source: Nature. 2018 Oct 24;562(7728):583–8. doi: 10.1038/s41586-018-0617-x (PMC6415775; doi:10.1038/s41586-018-0617-x)
Supplement: Supplementary file 1 — This file contains Supplementary Notes 1-3, Supplementary References and a full list of members of the TEDDY Study Group [file 41586_2018_617_MOESM1_ESM.pdf]

nature

SUPPLEMENTARY INFORMATION

https://doi.org/10.1038/s41586-018-0617-x

In the format provided by the authors and unedited.

Temporal development of the gut microbiome in early childhood from the TEDDY study

Christopher J. Stewart[1,2,18*], Nadim J. Ajami[1,18], Jacqueline L. O'Brien[1], Diane S. Hutchinson[1], Daniel P. Smith[1], Matthew C. Wong[1], Matthew C. Ross[1], Richard E. Lloyd[1], HarshaVardhan Doddapaneni[3], Ginger A. Metcalf[3], Donna Muzny[3], Richard A. Gibbs[3], Tommi Vatanen[4], Curtis Huttenhower[4], Ramnik J. Xavier[4], Marian Rewers[5], William Hagopian[6], Jorma Toppari[7,8], Anette-G. Ziegler[9,10,11], Jin-Xiong She[12], Beena Akolkar[13], Ake Lernmark[14], Heikki Hyoty[15,16], Kendra Vehik[17], Jeffrey P. Krischer[17] & Joseph F. Petrosino[1*]

[1]Alkek Center for Metagenomics and Microbiome Research, Department of Molecular Virology and Microbiology, Baylor College of Medicine, Houston, TX, USA. [2]Institute of Cellular Medicine, Newcastle University, Newcastle upon Tyne, UK. [3]Human Genome Sequencing Center, Baylor College of Medicine, Houston, TX, USA. [4]Broad Institute of MIT and Harvard, Cambridge, MA, USA. [5]Barbara Davis Center for Childhood Diabetes, University of Colorado, Aurora, CO, USA. [6]Pacific Northwest Research Institute, Seattle, WA, USA. [7]Institute of Biomedicine, Research Centre for Integrative Physiology and Pharmacology, University of Turku, Turku, Finland. [8]Department of Pediatrics, Turku University Hospital, Turku, Finland. [9]Institute of Diabetes Research, Helmholtz Zentrum München, Munich, Germany. [10]Forschergruppe Diabetes, Technische Universität München, Klinikum Rechts der Isar, Munich, Germany. [11]Forschergruppe Diabetes e.V. at Helmholtz Zentrum München, Munich, Germany. [12]Center for Biotechnology and Genomic Medicine, Medical College of Georgia, Augusta University, Augusta, GA, USA. [13]National Institute of Diabetes & Digestive & Kidney Diseases, Bethesda, MD, USA. [14]Department of Clinical Sciences, Lund University/CRC, Skane University Hospital, Malmö, Sweden. [15]Department of Virology, Faculty of Medicine and Biosciences, University of Tampere, Tampere, Finland. [16]Fimlab Laboratories, Pirkanmaa Hospital District, Tampere, Finland. [17]Health Informatics Institute, Morsani College of Medicine, University of South Florida, Tampa, FL, USA. [18]These authors contributed equally: Christopher J. Stewart, Nadim J. Ajami. *e-mail: christopher.stewart@ncl.ac.uk; jpetrosi@bcm.edu

© 2018 Springer Nature Limited. All rights reserved.

Temporal Development of the Gut Microbiome in Early Childhood - The TEDDY Study

Christopher J. Stewart, Nadim J. Ajami, Jacqueline L. O'Brien, Diane S. Hutchinson, Daniel P. Smith, Matthew C. Wong, Matthew C. Ross, Richard E. Lloyd, HarshaVardhan Doddapaneni, Ginger A. Metcalf, Donna Muzny, Richard A. Gibbs, Tommi Vatanen, Curtis Huttenhower, Ramnik J. Xavier, Marian Rewers, William Hagopian, Jorma Toppari, Anette G Ziegler, Jin-Xiong She, Beena Akolkar, Ake Lernmark, Heikki Hyoty, Kendra Vehik, Jeffrey P. Krischer, the TEDDY Study Group, and Joseph F. Petrosino

SUPPLEMENTRAY INFORMATION

This PDF contains:

Supplementary Note 1. Characterization of bacterial taxonomic and genetic capacity

Supplementary Note 2. Dirichlet multinomial mixtures (DMM) clustering of metagenomic sequencing data

Supplementary Note 3. Additional analysis of significant covariates association with the microbiome in early life

References for Supplementary Notes

Members of the TEDDY Study Group

Other Supplementary Material includes:

Supplementary Tables 1-4

SUPPLEMENTRAY NOTES

Supplementary Note 1. Characterization of bacterial taxonomic and genetic capacity

In months 3-7, 71% of samples were from infants receiving exclusive breast milk, exclusive formula, or a combination of breast milk and formula (**Extended Data Figure 1a**). From months 4-10, solid foods were introduced alongside breast milk and/or formula and only 9% of samples were from infants still not receiving solids at the time of sample collection. By 8.3 months, all samples were from infants receiving solid foods and by month 18, 88% of samples were from infants with a solids only diet (**Extended Data Figure 1a**). Bacterial richness (number of operational taxonomic units; OTUs) and diversity (Shannon index) increased rapidly over the first year of life, before stabilizing and increasing only marginally thereafter (**Extended Data Figure 1b**). Months 3 to 12 were characterised by declining Actinobacteria (average relative abundance of 53% at month 3 to 27% at month 12) and Proteobacteria (15% to 6%), coupled to increased Firmicutes (26% to 55%) (**Extended Data Figure 1c**). Bifidobacteria accounted for 97% of all Actinobacteria, with *B. longum*, *B. bifidum*, and *B. breve* predominantly accounting for the reduced temporal relative abundance within this phylum. From 20 months of life and onward, Actinobacteria (~21% relative abundance), Proteobacteria (~4%), and Firmicutes (~61%) remained stable. The abundance of Bacteroidetes and Verrucomicrobia increased gradually from month 3-40 of life to ~10% and ~3% relative abundance, respectively. Despite the dynamic nature of bacterial taxa, the metabolic capacity remained relatively consistent throughout sampling, especially after month 20 (**Extended Data Figure 1d**). ATP-binding cassette (ABC) transporters and two-component system (TCS) showed the greatest change, with both pathways showing comparable decreases in relative abundance from 3 to 20 months of life and remaining stable thereafter. ABC-transporter and TCS are involved in detection and signalling, such as detoxification peptide antibiotics produced by other resident bacteria in the gut microbiome [1]. ABC transporter gene expression is also regulated by a TCS in response to sensing a signal and the simultaneous decline in these pathways suggests that microbial sensing may be most important during early life, when the gut microbiome is at its

most dynamic. The stable metabolic capacity after the month 20 of life is comparable to data from adult populations [2]. This suggests that metabolic functions in declining species are replaced by other species in the microbiome.

Supplementary Note 2. Dirichlet multinomial mixtures (DMM) clustering of metagenomic sequencing data

The DMM modelling was also performed on the metagenomic sequencing data. Running DMM modelling on this dataset found microbiome profiles formed 18 unique clusters (based on lowest Laplace approximation) (**Extended Data Figure 2a-b**). The metagenomic sequencing analysis validated the three microbiome phases as described based on 16S rRNA gene sequencing data (**Extended Data Figure 2c**). In both datasets, *Bifidobacterium* dominated clusters in the developmental phase, *Tyzzerella* and *Eggerthella* dominated clusters in the transitional phase, and *Faecalibacterium* and *Ruminococcus* dominated clusters in the stable phase. The exact age when the infant microbiome reaches maturity has not been determined, but has been suggested to transition toward an adult-like profile between 1-3 years of life [3,4]. While the exact age is likely to vary between individuals, in the TEDDY population the infant microbiome was increasingly personalized and mature over the first year of life. After year one of life, individuals had a more specific and consistent microbiome signature, with notable dominance of taxa within Ruminococcaceae, Lachnospiraceae, Bacteroidaceae, and Verrucomicrobiaceae. The increased stability and specific abundant bacteria observed after month 31 of life is more comparable to data from adult populations [4].

Supplementary Note 3. Additional analysis of significant covariates association with the microbiome in early life

We performed analysis of covariates associated with modifying the development of the gut microbiome from 3 to 40 months of age, employing 16S rRNA gene sequencing (at the genus level) and whole genome shotgun metagenomics (at the species and functional level). In general, sporadic but statistically significant associations with a number of covariates including maternal, perinatal, gender, diet, supplements, environmental, and clinical factors were observed during different developmental stages. While each of these factors was not necessarily consistent in their significance of association by EnvFit when projected by genus, species, or functional metagenomes, they bear further mention nonetheless. Consistent with the companion manuscript by Vatanen *et al.*, at the functional level only breast milk feeding retained significance by EnvFit modelling (Figure 2c).

Breast-feeding was the most significant variable associated with gut microbiome development and is discussed in detail in the main manuscript text. Here, we perform additional analysis of all covariates that were significant at multiple time points and/or consistently significant by 16S rRNA gene sequencing and metagenomics. This corresponded to birth mode, geographical location, living with furry pets, living with siblings, and infant probiotic use. As infant probiotic use is analysed in the companion Vatanen et al. manuscript, we focus here on the other key variables.

Birth mode shaped the development of the microbiome through the first year of life, driven by *Bacteroides* sp.

Birth mode was significantly associated with the gut microbiome over the first year of life (**Supplementary Table 1**). Despite the high rate of maternal T1D (7%) and gestational diabetes (6%), this covariate was not associated with offspring microbiome profiles at any time point. *Bacteroides* and *Parabacteroides* showed higher relative abundance in vaginally delivered infants, whereas *Enterococcus* and *Clostridium* were higher in caesarian delivered infants (**Supplementary Table 2 and Extended Data Figure 5a**). A total of 29 species were significantly different between caesarian and vaginal delivery between 3 to 14 months of life, of which 18 *Bacteroides* spp. and 6

Parabacteroides spp. were higher in vaginally delivery infants (full list of significant taxa and associated P values presented in **Supplementary Table 2**). *B. vulgatus*, *B. fragilis*, *B. thetaiotaomicron*, *B. xylanisolvens, B. ovatus,* and *B. uniformis* were the most abundant *Bacteroides* spp. and were all significantly higher in vaginal infants throughout the first year of life. *B. fragilis* showed especially stark delayed establishment in caesarian infants from 3 to 10 months of life (**Extended Data Figure 5b**).

It has been suggested that ~20% of vaginally delivered infants lack *Bacteroides* and are thus more comparable to caesarian infants over the first year of life [5]. We extended the birth mode analysis further to explore this and classified samples as positive (any *Bacteroides* reads) or negative (no *Bacteroides* reads). At months 3-6, 29% of vaginal infants were positive for *Bacteroides*, compared to 54% of caesarian infants (P <0.001). Samples within vaginal and caesarian groups clustered distinctly, with samples increasingly clustering by *Bacteroides* status through the early time points, regardless of birth mode (**Extended Data Figure 5c**). Furthermore, the presence of *Bacteroides* drove microbiome development, with *Bacteroides* positive samples associated with increased temporal richness and diversity, regardless of vaginal or caesarian birth (**Extended Data Figure 5d**). Specifically, the temporal richness (developmental phase P = 0.539, transitional phase P = 0.606, and stable phase P = 0.470) and diversity (developmental phase P = 0.894, transitional phase P = 0.962, and stable phase P = 0.730) was not significantly different between birth modes. However, *Bacteroides* positive samples had significantly increased richness in the developmental phase only (developmental phase P <0.001, transitional phase P = 0.129, and stable phase P = 0.063) and significantly higher diversity in all phases (developmental phase P = 0.004, transitional phase P = 0.009, and stable phase P = 0.002), compared to that in samples without *Bacteroides* (**Extended Data Figure 5d**). Thus, while vaginally delivered infants are more likely to be colonised by *Bacteroides*, if an infant born by caesarian has detectable *Bacteroides*, they will show comparable microbiome development to the *Bacteroides* positive vaginal infants. By the start of the transitional phase at months 15-18, the majority of samples were *Bacteroides*

positive and comparable between birth mode groups, where 91% of vaginal and 87% of caesarian infants had detectable *Bacteroides*. By the start of the stable phase at month 31, 94% of vaginal and 97% of caesarian infants had detectable *Bacteroides*.

For microbiota age and MAZ, caesarean infants had significantly increased maturation in the developmental phase (microbiota age P = 0.003 and MAZ P = 0.002) and no significance was found in the transitional phase (P = 0.200 and P = 0.146) (**Extended Data Figure 5e**). Despite the initial delayed microbiome maturation, vaginal infants had significantly increased maturation during the stable phase when compared to caesarean infants (P <0.001 for both microbiota age and MAZ). Detectable *Bacteroides* was also associated with increased microbiome maturation throughout sampling, reaching significance in the developmental phase (microbiota age P = 0.031 and MAZ P = 0.013; **Extended Data Figure 5e**).

No covariate explained why some vaginal infants had detectable *Bacteroides* and others did not. For instance, the detection of *Bacteroides* in vaginal infants was not explained by breast milk status (**Extended Data Figure 6a**). Infants who never received breast milk had elevated *Bacteroides*, but low numbers of infants (*n* = 17, 9 vaginal and 8 caesarian) precluded robust statistical comparisons of this group. The changes in relative abundance of the six most dominant *Bacteroides* spp. were consistent between each geographical location (**Extended data Figure 6b**) and none of the dominant *Bacteroides* spp. were significantly associated geographical location at any of the time windows (**Supplementary Table 2**). However, while the increase in *Bacteroides* relative abundance existed across 5/6 geographical locations, opposing trends were found in infants from Washington (**Extended data Figure 6c**). This is in line with published data from single locations, with existing studies primarily supporting overall increased *Bacteroides* in vaginally delivered infants [5–10], but some studies (and hence locations) finding no associations [11,12]. Thus, the location of single geographical cohorts may account for why some studies fail to find the *Bacteroides* association, underscoring the importance of multi-geographical cohorts.

The abundance of *Bacteroides* in the cohort is also likely to have important consequences when comparing birth mode. While we adjusted for maternal and post-natal factors that may influence *Bacteroides* abundance, it is possible that the differences observed within the caesarian population reflect unavailable metadata, such as the underlying clinical indication for caesarian or a laboured birth [11,12]. In line with the findings in the current study, existing work has shown that maternal Bacteroidia strains are only transmitted in vaginal deliveries, whereas caesarian infants gradually acquire maternal Bacteroidia strains over the first year of life [13]. Thus, direct transmission of *Bacteroides* from the mother at birth during vaginal delivery may account for the overall increased *Bacteroides* spp. over the first year of life.

Geographical location and household exposures contribute to microbiome development in early life

TEDDY recruitment locations are all typical of Western populations (e.g., comparable clinical practice, sanitation, culture, etc.) and 62% of known ethnicity reported as non-Hispanic white (**Extended Data Table 1**). It has been suggested that US and European populations show comparable microbiome structure, but developing nations are distinct from Western populations [14]. In the current cohort, differences between specific geographical locations occurred from 3-22 months of life (**Supplementary Table 1**). Species associated with geographical location were found sporadically across time points (**Supplementary Table 2**) and diversity, microbiota age, and MAZ had comparable trajectories across each geographical location (**Extended Data Figure 7a-c**). The prevalence of bacterial genera, also termed the core microbiome (genera present in >90% of samples), was comparable between geographical locations through each of the three phases (**Supplementary Table 4**). With the exception of Washington in the developmental phase, *Bifidobacterium* was a found in the core microbiome in all locations at all phases. As well as *Bifidobacterium*, *Ruminococcus* gnavus group and *Erysipelatoclostridium* were core genera in all six locations during the transitional phase (**Supplementary Table 4**). These genera were also core

in all locations during the stable phase, along with *Bacteroides*, *Anaerostipes*, *Blautia*, Lachnospiraceae UCG-008, and *Intestinibacter* (**Supplementary Table 4**).

The impact of household exposures (e.g., siblings and pets) on infant microbiome development has received growing interest over recent years, owing to the potential influence of these factors in developing allergic diseases and asthma [15–18]. In the current study, 57% of infants were living with at least one sibling (**Extended Data Table 1**) and this covariate was significantly associated with microbiome profiles from month 3 to 18 of life (**Figure 2**). While the temporal longitudinal bacterial diversity was comparable (**Extended Data Figure 7d**), months 15-30 (i.e., the transitional phase) accounted for the vast majority of significantly altered bacterial taxa (**Supplementary Table 2**). During this period, in months 15-18, *F. prausnitzii* (P = 0.001), *B. adolescentis* (P = 0.001), *Eubacterium siraeum* (P <0.001) and *Dorea formicigenerans* (P = 0.001) were most significantly associated with infants with living siblings (**Supplementary Table 2**). These taxa were highest in the latter months and infants' living with sibling showed accelerated microbiome maturation, however, this maturation did not reach significance (P = 0.073; **Extended Data Figure 7e-f**). While the concurrent sampling of siblings and homes was not performed in the current study, existing data has demonstrated that cohabiting family members can share microbiota with each other [19], providing a likely mechanism for the reported difference.

Living with furry pets also influenced the gut microbiome up to month 10 of life, similar to recent works, including the CHILD cohort (Canadian infants) [20,21]. In the current study, cohabiting with furry pets, represented primarily by a cat and/or dog (90% of all furry positive samples), was significantly associated with the microbiome by 16S rRNA gene sequencing at months 3-6 (**Supplementary Table 2**). *Bifidobacterium* was the most significantly altered taxa and was reduced in infants with furry pets, supporting existing data [21]. Accordingly, *Bifidobacterium* was abundant in early life samples and during the developmental phase living with furry pets was associated with significantly increased diversity (P = 0.021), microbiota age (P <0.001), and MAZ (P <0.001). Furthermore, living with furry pets was not found to significantly alter the diversity,

microbiota age, or MAZ in either the transitional or stable phases (P > 0.05; **Extended Data Figure 7g-i**). Given the stochasticity of the infant exposures during development and the highly individual nature of the microbiome between humans and animals, it is intriguing that influences of household exposures (e.g., sibling and pets) are reproducibly found across studies (and thus different populations).

References

1. Staroń, A., Finkeisen, D. E. & Mascher, T. Peptide antibiotic sensing and detoxification modules of Bacillus subtilis. *Antimicrob. Agents Chemother.* **55,** 515–525 (2011).
2. Human, T. & Project, M. Structure, function and diversity of the healthy human microbiome. *Nature* **486,** 207–14 (2012).
3. Yatsunenko, T. *et al.* Human gut microbiome viewed across age and geography. *Nature* (2012). doi:10.1038/nature11053
4. Lozupone, C. A. *et al.* Meta-analyses of studies of the human microbiota. *Genome Res.* **23,** 1704–1714 (2013).
5. Yassour, M. *et al.* Natural history of the infant gut microbiome and impact of antibiotic treatment on bacterial strain diversity and stability. *Sci. Transl. Med.* **8,** 343–81 (2016).
6. Bokulich, N. A. *et al.* Antibiotics, birth mode, and diet shape microbiome maturation during early life. *Sci. Transl. Med.* **8,** 1–14 (2016).
7. Bäckhed, F. *et al.* Dynamics and Stabilization of the Human Gut Microbiome during the First Year of Life. *Cell Host Microbe* **17,** 690–703 (2015).
8. Hesla, H. M. *et al.* Impact of lifestyle on the gut microbiota of healthy infants and their mothers - the ALADDIN birth cohort. *FEMS Microbiol. Ecol.* **90,** 791–801 (2014).
9. Gregory, K. E., LaPlante, R. D., Shan, G., Kumar, D. V. & Gregas, M. Mode of Birth Influences Preterm Infant Intestinal Colonization With Bacteroides Over the Early Neonatal Period. *Adv. Neonatal Care* **15,** 386–393 (2015).
10. Penders, J. *et al.* Factors influencing the composition of the intestinal microbiota in early infancy. *Pediatrics* **118,** 511–21 (2006).
11. Chu, D. M. *et al.* Maturation of the infant microbiome community structure and function across multiple body sites and in relation to mode of delivery. *Nat. Med.* **23,** 314–326 (2017).
12. Azad, M. B. *et al.* Gut microbiota of healthy Canadian infants: profiles by mode of delivery and infant diet at 4 months. *CMAJ* **185,** 385–94 (2013).
13. Korpela, K. *et al.* Selective maternal seeding and environment shape the human gut microbiome. *Genome Res.* **in press,** gr.233940.117 (2018).
14. Arrieta, M.-C., Stiemsma, L. T., Amenyogbe, N., Brown, E. M. & Finlay, B. The Intestinal Microbiome in Early Life: Health and Disease. *Front. Immunol.* **5,** 427 (2014).
15. Ball, T. M. *et al.* Siblings, Day-Care Attendance, and the Risk of Asthma and Wheezing during Childhood. *N. Engl. J. Med.* **343,** 538–543 (2000).
16. Koplin, J. J. *et al.* Environmental and demographic risk factors for egg allergy in a population-based study of infants. *Allergy* **67,** 1415–1422 (2012).
17. Benn, C. S., M, M., Wohlfahrt, J., Björkstén, B. & Aaby, P. Cohort study of sibling effect, infectious diseases, and risk of atopic dermatitis during first 18 months of life. *BMJ* **328,** 1223–0 (2004).
18. Hesselmar, B., Åberg, N., Åberg, B., Eriksson, B. & Björkstén, B. Does early exposure to cat

or dog protect against later allergy development? *Clin. Exp. Allergy* **29,** 611–617 (1999).
19. Song, S. J. *et al.* Cohabiting family members share microbiota with one another and with their dogs. *Elife* **2,** e00458 (2013).
20. Tun, H. M. *et al.* Exposure to household furry pets influences the gut microbiota of infant at 3–4 months following various birth scenarios. *Microbiome* **5,** 40 (2017).
21. Azad, M. B. *et al.* Infant gut microbiota and the hygiene hypothesis of allergic disease: impact of household pets and siblings on microbiota composition and diversity. *Allergy, Asthma Clin. Immunol.* **9,** 15 (2013).

The TEDDY Study Group

Colorado Clinical Center: Marian Rewers, M.D., Ph.D., PI[1,4,5,6,10,11], Kimberly Bautista[12], Judith Baxter[9,10,12,15], Ruth Bedoy[2], Daniel Felipe-Morales, Kimberly Driscoll, Ph.D.[9], Brigitte I. Frohnert, M.D.[2,14], Marisa Gallant, M.D.[13], Patricia Gesualdo[2,6,12,14,15], Michelle Hoffman[12,13,14], Rachel Karban[12], Edwin Liu, M.D.[13], Jill Norris, Ph.D.[2,3,12], Adela Samper-Imaz, Andrea Steck, M.D.[3,14], Kathleen Waugh[6,7,12,15], Hali Wright[12]. University of Colorado, Anschutz Medical Campus, Barbara Davis Center for Childhood Diabetes.

Finland Clinical Center: Jorma Toppari, M.D., Ph.D., PI[¥^1,4,11,14], Olli G. Simell, M.D., Ph.D., Annika Adamsson, Ph.D.[^12], Suvi Ahonen*[±§], Heikki Hyöty, M.D., Ph.D.*[±6], Jorma Ilonen, M.D., Ph.D.[¥¶3], Sanna Jokipuu[^], Tiina Kallio[^], Leena Karlsson[^], Miia Kähönen[µ¤], Mikael Knip, M.D., Ph.D.*[±5], Lea Kovanen*[±§], Mirva Koreasalo*[±§2], Kalle Kurppa, M.D., Ph.D.*[±13], Tiina Latva-aho[µ¤], Maria Lönnrot, M.D., Ph.D.*[±6], Elina Mäntymäki[^], Katja Multasuo[µ¤], Tiina Niininen[±]*[12], Sari Niinistö[±§2], Mia Nyblom*[±], Petra Rajala[^], Jenna Rautanen[±§], Anne Riikonen*[±§], Minna Romo[^], Juulia Rönkä[µ¤], Satu Simell, M.D., Ph.D.[¥13], Tuula Simell, Ph.D.[¥12], Maija Sjöberg[¥^12,14], Aino Stenius[µ¤12], Maria Leppänen[^], Sini Vainionpää[^], Eeva Varjonen[¥^12], Riitta Veijola, M.D., Ph.D.[µ¤14], Suvi M. Virtanen, M.D., Ph.D.*[±§2], Mari Vähä-Mäkilä[^], Mari Åkerlund*[±§], Katri Lindfors, Ph.D.*[13] [¥]University of Turku, *University of Tampere, [µ]University of Oulu, [^]Turku University Hospital, Hospital District of Southwest Finland, [±]Tampere University Hospital, [¤]Oulu University Hospital, §National Institute for Health and Welfare, Finland, [¶]University of Kuopio.

Georgia/Florida Clinical Center: Jin-Xiong She, Ph.D., PI[1,3,4,11], Desmond Schatz, M.D.*[4,5,7,8], Diane Hopkins[12], Leigh Steed[12,13,14,15], Jennifer Bryant, Jamie Thomas*[6,12], Janey Adams*[12], Katherine Silvis[2], Michael Haller, M.D.*[14], Melissa Gardiner, Richard McIndoe, Ph.D., Ashok Sharma, Stephen W. Anderson, M.D.[^], Laura Jacobsen, M.D.[*14] Center for Biotechnology and Genomic Medicine, Augusta University. *University of Florida, [^]Pediatric Endocrine Associates, Atlanta.

Germany Clinical Center: Anette G. Ziegler, M.D., PI[1,3,4,11], Andreas Beyerlein, Ph.D.[2], Ezio Bonifacio Ph.D.*[5], Anja Heublein, Michael Hummel, M.D.[13], Sandra Hummel, Ph.D.[2], Annette Knopff[7], Charlotte Koch, Sibylle Koletzko, M.D.[¶13], Claudia Ramminger, Roswith Roth, Ph.D.[9], Marlon Scholz, Laura Schulzik[2], Joanna Stock[9,12,14], Katharina Warncke, M.D.[14], Lorena Wendel, Christiane Winkler, Ph.D.[2,12,15]. Forschergruppe Diabetes e.V. and Institute of Diabetes Research, Helmholtz Zentrum München, Forschergruppe Diabetes, and Klinikum rechts der Isar, Technische Universität München. *Center for Regenerative Therapies, TU Dresden, [¶]Dr. von Hauner Children's Hospital, Department of Gastroenterology, Ludwig Maximillians University Munich.

Sweden Clinical Center: Åke Lernmark, Ph.D., PI[1,3,4,5,6,8,10,11,15], Daniel Agardh, M.D., Ph.D.[13], Carin Andrén Aronsson, Ph.D.[2,12,13], Maria Ask, Jenny Bremer, Ulla-Marie Carlsson, Corrado Cilio, Ph.D., M.D.[5], Emelie Ericson-Hallström, Annika Fors, Lina Fransson, Thomas Gard, Rasmus Bennet, Carina Hansson, Susanne Hyberg, Hanna Jisser, Fredrik Johansen, Berglind Jonsdottir, M.D., Silvija Jovic, Helena Elding Larsson, M.D., Ph.D. [6,14], Marielle Lindström, Markus Lundgren, M.D.[14], Maria Månsson-Martinez, Maria Markan, Jessica Melin[12], Zeliha Mestan, Caroline Nilsson, Karin Ottosson, Kobra Rahmati, Anita Ramelius, Falastin Salami, Sara Sibthorpe, Anette Sjöberg, Birgitta Sjöberg, Evelyn Tekum Amboh, Carina Törn, Ph.D. [3,15], Anne Wallin, Åsa Wimar[14], Sofie Åberg. Lund University.

Washington Clinical Center: William A. Hagopian, M.D., Ph.D., PI[1,3,4, 5, 6,7,11,13, 14], Michael Killian[6,7,12,13], Claire Cowen Crouch[12,14,15], Jennifer Skidmore[2], Ashley Akramoff, Jana Banjanin, Masumeh Chavoshi, Kayleen Dunson, Rachel Hervey, Shana Levenson, Rachel Lyons, Arlene Meyer, Denise Mulenga, Davey Schmitt, Julie Schwabe. Pacific Northwest Research Institute.

Pennsylvania Satellite Center: Dorothy Becker, M.D., Margaret Franciscus, MaryEllen Dalmagro-Elias Smith[2], Ashi Daftary, M.D., Mary Beth Klein, Chrystal Yates. Children's Hospital of Pittsburgh of UPMC.

Data Coordinating Center: Jeffrey P. Krischer, Ph.D.,PI[1,4,5,10,11], Sarah Austin-Gonzalez, Maryouri Avendano, Sandra Baethke, Rasheedah Brown[12,15], Brant Burkhardt, Ph.D.[5,6], Martha Butterworth[2], Joanna Clasen, David Cuthbertson, Christopher Eberhard, Steven Fiske[9], Dena Garcia, Jennifer Garmeson, Veena Gowda, Kathleen Heyman, Belinda Hsiao, Francisco Perez Laras, Hye-Seung Lee, Ph.D.[1,2,13,15], Shu Liu, Xiang Liu, Ph.D.[2,3,9,14], Kristian Lynch, Ph.D. [5,6,9,15], Colleen Maguire, Jamie Malloy, Cristina McCarthy[12,15], Aubrie Merrell, Steven Meulemans, Hemang Parikh, Ph.D.[3], Ryan Quigley, Cassandra Remedios, Chris Shaffer, Laura Smith, Ph.D.[9,12], Susan Smith[12,15], Noah Sulman, Ph.D., Roy Tamura, Ph.D.[1,2,13], Ulla Uusitalo, Ph.D.[2,15], Kendra Vehik, Ph.D.[4,5,6,14,15], Ponni Vijayakandipan, Keith Wood, Jimin Yang, Ph.D., R.D.[2,15]. *Past staff: Michael Abbondondolo, Lori Ballard, David Hadley, Ph.D., Wendy McLeod.* University of South Florida.

Project scientist: Beena Akolkar, Ph.D.[1,3,4,5,6,7,10,11]. National Institutes of Diabetes and Digestive and Kidney Diseases.

Autoantibody Reference Laboratories: Liping Yu, M.D.[^5], Dongmei Miao, M.D.[^], Polly Bingley, M.D., FRCP*[5], Alistair Williams*, Kyla Chandler*, Claire Williams*, Gifty George*, Sian Grace*, Ben Gillard*. [^]Barbara Davis Center for Childhood Diabetes, University of Colorado Denver, *Bristol Medical School, University of Bristol UK.

HLA Reference Laboratory: William Hagopian[3], MD, PhD, Masumeh Chavoshi, Pacific Northwest Research Institute, Seatte WA. (Previously Henry Erlich, Ph.D.[3], Steven J. Mack, Ph.D., Anna Lisa Fear. Center for Genetics, Children's Hospital Oakland Research Institute.)

Metagenomics and Microbiome Laboratory: Joseph F. Petrosino, Ph.D.[6*], Nadim J. Ajami, Ph.D.[*], Richard E. Lloyd, Ph.D.[6*], Matthew C. Ross, Ph.D.[*], Jacqueline L. O'Brien, Ph.D.[*], Diane S. Hutchinson, Ph.D.[*], Daniel P. Smith, Ph.D.[*], Matthew C. Wong[*], Xianjun Tian, Ph.D.[*], Tulin Ayvaz[*], Auriole Tamegnon[*], Nguyen Truong[*], Hannah Moreno[*], Lauren Riley[*], Eduardo Moreno[*], Tonya Bauch[*], Lenka Kusic[*], Ginger Metcalf[^], Donna Muzny[^], HarshaVArdhan Doddapaneni, Ph.D.[^], Richard Gibbs, Ph.D.[^]. *Alkek Center for Metagenomics and Microbiome Research, Department of Molecular Virology and Microbiology, Baylor College of Medicine, ^Human Genome Sequencing Center, Baylor College of Medicine.

Repository: Sandra Ke, Niveen Mulholland, Ph.D. NIDDK Biosample Repository at Fisher BioServices.

Other contributors: Kasia Bourcier, Ph.D.[5], National Institutes of Allergy and Infectious Diseases. Thomas Briese, Ph.D.[6,15], Columbia University. Suzanne Bennett Johnson, Ph.D.[9,12], Florida State University. Eric Triplett, Ph.D.[6], University of Florida.

Committees:
[1]Ancillary Studies, [2]Diet, [3]Genetics, [4]Human Subjects/Publicity/Publications, [5]Immune Markers, [6]Infectious Agents, [7]Laboratory Implementation, [8]Maternal Studies, [9]Psychosocial, [10]Quality Assurance, [11]Steering, [12]Study Coordinators, [13]Celiac Disease, [14]Clinical Implementation, [15]Quality Assurance Subcommittee on Data Quality.
