# Supplementary material for: Temporal development of the gut microbiome in early childhood from the TEDDY study
Source: Nature. 2018 Oct 24;562(7728):583–8. doi: 10.1038/s41586-018-0617-x (PMC6415775; doi:10.1038/s41586-018-0617-x)
Supplement: Supplementary file 2 — Reporting Summary [file 41586_2018_617_MOESM2_ESM.pdf]

nature research

Corresponding author(s): Christopher J. Stewart
Joseph F. Petrosino

Reporting Summary

Nature Research wishes to improve the reproducibility of the work that we publish. This form provides structure for consistency and transparency in reporting. For further information on Nature Research policies, see Authors & Referees and the Editorial Policy Checklist.

Statistical parameters

When statistical analyses are reported, confirm that the following items are present in the relevant location (e.g. figure legend, table legend, main text, or Methods section).

n/a	Confirmed	
☐	☒	The exact sample size (*n*) for each experimental group/condition, given as a discrete number and unit of measurement
☐	☒	An indication of whether measurements were taken from distinct samples or whether the same sample was measured repeatedly
☐	☒	The statistical test(s) used AND whether they are one- or two-sided<br>*Only common tests should be described solely by name; describe more complex techniques in the Methods section.*
☐	☒	A description of all covariates tested
☐	☒	A description of any assumptions or corrections, such as tests of normality and adjustment for multiple comparisons
☐	☒	A full description of the statistics including central tendency (e.g. means) or other basic estimates (e.g. regression coefficient) AND variation (e.g. standard deviation) or associated estimates of uncertainty (e.g. confidence intervals)
☐	☒	For null hypothesis testing, the test statistic (e.g. *F*, *t*, *r*) with confidence intervals, effect sizes, degrees of freedom and *P* value noted<br>*Give P values as exact values whenever suitable.*
☒	☐	For Bayesian analysis, information on the choice of priors and Markov chain Monte Carlo settings
☐	☒	For hierarchical and complex designs, identification of the appropriate level for tests and full reporting of outcomes
☐	☒	Estimates of effect sizes (e.g. Cohen's *d*, Pearson's *r*), indicating how they were calculated
☐	☒	Clearly defined error bars<br>*State explicitly what error bars represent (e.g. SD, SE, CI)*

Our web collection on statistics for biologists may be useful.

Software and code

Policy information about availability of computer code

Data collection	No software was used for data collection.
Data analysis	USEARCH v7.0.1090<br>UCHIME v4.2<br>Bowtie2 v2.2.3<br>Casava v1.8.2<br>cutadapt v1.9dev2<br>Trim Galore 0.2.8<br>PRINSEQ v0.20.3<br>MinPath v1.2<br>Analysis was performed in R v3.3.1 in R Studio v1.0.136<br>R vegan package 2.4-2<br>R ggplot package 2_2.2.1<br>R randomForest package 4.6-12<br>R lme4 package 1.1-13<br>MaAsLin 0.0.4

For manuscripts utilizing custom algorithms or software that are central to the research but not yet described in published literature, software must be made available to editors/reviewers upon request. We strongly encourage code deposition in a community repository (e.g. GitHub). See the Nature Research guidelines for submitting code & software for further information.

Data

Policy information about availability of data

All manuscripts must include a data availability statement. This statement should provide the following information, where applicable:

- Accession codes, unique identifiers, or web links for publicly available datasets
- A list of figures that have associated raw data
- A description of any restrictions on data availability

TEDDY Microbiome 16S and WGS data that support the findings of this study will be made available in NCBI's database of Genotypes and Phenotypes (dbGaP) with the primary accession code phs001443.v1, in accordance with the dbGaP controlled-access authorization process.

Clinical metadata analyzed during the current study will be made available in the NIDDK Central Repository at https://www.niddkrepository.org/studies/teddy

Field-specific reporting

Please select the best fit for your research. If you are not sure, read the appropriate sections before making your selection.

☒ Life sciences ☐ Behavioural & social sciences ☐ Ecological, evolutionary & environmental sciences

For a reference copy of the document with all sections, see nature.com/authors/policies/ReportingSummary-flat.pdf

Life sciences study design

All studies must disclose on these points even when the disclosure is negative.

Sample size	Longitudinal stool samples between months 3-46 of life from 903 children were analyzed by 16S rRNA gene sequencing (n=12,005) and metagenomic sequencing (n=10,867). The cohort is a nested case-control design.
Data exclusions	For the nested case-control analysis, some samples were removed so that exactly the same number of samples was included between cases and controls. This prevented skewing data due to the generally increased number of samples from cases
Replication	Observational cohort. No replication
Randomization	Controls were matched individually to cases as described in detail in the manuscript text. Cases were sampled until diagnosis of T1D and matched control samples were included up until the corresponding day of life.
Blinding	No blinding used, TEDDY is an observational follow-up study

Reporting for specific materials, systems and methods

Materials & experimental systems

n/a	Involved in the study	
☐	☒	Unique biological materials
☒	☐	Antibodies
☒	☐	Eukaryotic cell lines
☒	☐	Palaeontology
☒	☐	Animals and other organisms
☐	☒	Human research participants

Methods

n/a	Involved in the study	
☒	☐	ChIP-seq
☒	☐	Flow cytometry
☒	☐	MRI-based neuroimaging

Unique biological materials

Policy information about availability of materials

Obtaining unique materials	All stool sample material may have been used for DNA extraction. In some cases investigators may be able to access the DNA or sample from The TEDDY Study Group

Human research participants

Policy information about studies involving human research participants

Population characteristics	Children were aged 3-46 months for the analysis. Children samples were obtained from six geographical locations (Finland, Germany, Sweden in Europe and Washington, Colorado and Georgia in the United States). The cohort is at high risk for developing IA or T1D, with half of the cohort cases and the other half controls.
Recruitment	Children were recruited based on risk for T1D (e.g., parental history, HLA, etc.). This is described in depth in the methods.
